# PlmCas12e (CasX2) cleavage of CCR5: impact of guide RNA spacer length and PAM sequence on cleavage activity

**DOI:** 10.1080/15476286.2023.2221510

**Published:** 2023-06-07

**Authors:** David A. Armstrong, Taylor R. Hudson, Christine A. Hodge, Thomas H. Hampton, Alexandra L. Howell, Matthew S. Hayden

**Affiliations:** a Research Service, V.A. Medical Center, White River Junction, VT, USA; b Departments of Dermatology, Dartmouth Health, Lebanon, NH, USA; c Departments of Dermatology, Geisel School of Medicine at Dartmouth, Hanover, NH, USA; d Departments of Microbiology/Immunology, Geisel School of Medicine at Dartmouth, Hanover, NH, USA; e Departments of Medicine, Dartmouth Health, Lebanon, NH, USA

**Keywords:** Crispr/Cas, CasX2, CCR5, Guide RNA, PAM requirements

## Abstract

Gene editing using CRISPR/Cas (clustered regularly interspaced palindromic repeats/CRISPR-associated) is under development as a therapeutic tool for the modification of genes in eukaryotic cells. While much effort has focused on CRISPR/Cas9 systems from *Streptococcus pyogenes* and *Staphylococcus aureus*, alternative CRISPR systems have been identified from non-pathogenic microbes, including previously unknown class 2 systems, adding to a diverse toolbox of CRISPR/Cas enzymes. The Cas12e enzymes from non-pathogenic Deltaproteobacteria (CasX1, DpeCas12e) and Planctomycetes (CasX2, PlmCas12e) are smaller than Cas9, have a selective protospacer adjacent motif (PAM), and deliver a staggered cleavage cut with a 5–7 nucleotide overhang. We investigated the impact of guide RNA spacer length and alternative PAM sequences on cleavage activity to determine optimal conditions for PlmCas12e cleavage of the cellular gene *CCR5* (CC-Chemokine receptor-5). *CCR5* encodes the CCR5 coreceptor used by human immunodeficiency virus-type 1 (HIV-1) to infect target cells. A 32 base-pair deletion in *CCR5* (*CCR5-Δ32*) is responsible for HIV-1 resistance and reported cures following bone marrow transplantation. Consequently, *CCR5* has been an important target for gene editing utilizing CRISPR/Cas. We determined that *CCR5* cleavage activity varied with the target site, spacer length, and the fourth nucleotide in the previously described PAM sequence, TTCN. Our analyses demonstrated a PAM preference for purines (adenine, guanine) over pyrimidines (thymidine, cytosine) in the fourth position of the CasX2 PAM. This improved understanding of CasX2 cleavage requirements facilitates the development of therapeutic strategies to recreate the *CCR5-Δ32* mutation in haematopoietic stem cells.

## Introduction

The Cysteine-Cysteine Chemokine Receptor type 5 (CCR5) was identified as a critical co-receptor for target cell infection by macrophage-tropic primary isolates of human immunodeficiency virus type 1 (HIV-1) [1,2]. Since then, the *CCR5* gene or its protein receptor have been targeted for modification as an approach to inhibit or prevent HIV-1 infection of T lymphocytes and myeloid cells [3]. Early attempts to mutate or delete *CCR5* used TALENs (transcription activator-like effector nucleases) [4], or ZFNs (zinc finger nucleases) [5], as approaches to excise critical gene regions within the *CCR5* coding region [6–11]. Another approach to modify *CCR5* gene expression has used base editors [12,13] to alter the sequence of nucleotides in the gene sequence that leads to the insertion of targeted point mutations or to the introduction of premature stop codons.

More recently, genome editing using CRISPR/Cas (clustered regularly interspaced palindromic repeats/CRISPR-associated) has been used to achieve precise gene cleavage of both cellular and integrated viral genes in eukaryotic cells and in pre-clinical animal models [14–19]. This method provides a powerful approach to eliminate, modify, or replace specific genes or gene sequences, using a combination of a Cas endonuclease, and a short RNA molecule known as the guide RNA (gRNA). In the case of the well-characterized Cas9 systems, the gRNA is most often configured as a single gRNA (sgRNA). The sgRNA is a single, continuous strand of RNA comprised of a tracr-RNA (trans-activating CRISPR-RNA) with a flexible linkage to the crRNA (CRISPR-RNA) consisting of repeat and spacer sequences. The spacer binds in a complementary fashion to the target region of the DNA known as the protospacer, while the remaining portion of the sgRNA is involved in intramolecular interactions and intermolecular interactions with the Cas enzyme.

CRISPR/Cas gene editing has provided novel approaches for the development of a therapeutic to treat sickle cell disease (CTX001), HIV-1 (EBT-101), and non-small cell lung cancer [20], with several other CRISPR-based clinical trials in the pipeline. The key to CRISPR’s specificity lies in the binding of the spacer to the complementary protospacer region. The adjacent protospacer adjacent motif (PAM) serves to licence the Cas endonuclease activity. Target site selection is based on multiple parameters. For example, the presence of a PAM dictates the location and orientation of potential target sites. The DNA strand that contains the PAM sequence is referred to as the non-target strand (NTS), and the gRNA binds to the DNA strand without the PAM sequence, known as the target strand (TS). Further, the similarity of the target sequence to other genomic sites must also be considered to reduce the potential for off-target cleavage events. *CCR5* targeting is challenging in this regard, given the homology of *CCR5* to the *CCR2* gene [21] that encodes a related C-C chemokine receptor, CCR2. CRISPR/Cas cleavage of *CCR5* in haematopoietic cells was shown to cause loss of CCR5 receptor expression on differentiated progeny cells [22]. Targeting *CCR5* in haematopoietic stem cells as an approach to generate a CCR5^null^ immune system was supported by clinical reports of the permanent suppression of HIV replication in two different patients who underwent allogeneic bone marrow transplantation using bone marrow from donors homozygous for the *CCR5-Δ32* mutation [23–26]. To date, the most common sources of Cas endonucleases, Cas9, derive from the common human pathogens *Staphylococcus aureus* (SaCas9) and *Streptococcus pyogenes* (SpCas9) yet pre-existing immunity to these bacteria as well as the rather large molecular size of SpCas9 may limit the long-term utility of these Cas enzymes in therapeutic approaches [27–29].

Recent metagenomic sequencing and computational efforts have identified a wide variety of new CRISPR systems and have led to the discovery of Cas12a [30,31], Cas12b [32,33], Cas12d [34,35], Cas12e [35,36], and the RNA-cleaving Cas13 family [37]. Deltaproteobacteria Cas12e (DpbCas12e) and Planctomycetes Cas12e (PlmCas12e) were identified along with several Cas12d family members (CasY) from a metagenomic survey by Burstein et al. [35]. Further analyses of Cas12e enzymes demonstrated efficient RNA-guided cleavage using a 20 nucleotide (nt) crRNA complexed to a tracrRNA [38]. Cleavage of the DNA target occurred adjacent to a PAM sequence of ‘TTCN’ in which the first three bases of the PAM sequence, thymidine-thymidine-cytosine, were strictly required, while for the spacer tested, no preference was observed for a specific base at the fourth position, ‘N’, immediately adjacent to the protospacer [38]. Cleavage products were identified on both the TS and NTS in an asymmetrical (staggered) pattern characteristic of Cas enzymes with a single recombination UV (Ruv)C cleavage site [38].

We sought to define optimal conditions for PlmCas12e (CasX2) cleavage of DNA, using as a target, a 1,250 base pair (bp) fragment of *CCR5* [39] flanking the area that includes the region deleted in the *CCR5-Δ32* mutation [40]. Our interest in optimizing CasX2 cleavage stemmed from the potential to use this editor to replace the relevant wild-type region of *CCR5* with the *CCR5-Δ32* mutation. This could be accomplished by using carefully selected pairs of gRNAs that flank the region deleted in the *CCR5-Δ32* mutation, together with a donor DNA template lacking the critical 32 nucleotides and containing flanking homology arms. Such approaches would benefit from an optimized gRNA design to increase cutting efficiency and specificity. The longer 5’ overhangs resulting from CasX2 offset TS and NTS cleavage sites are predicted to promote homology-directed repair mechanisms in cells, based on experience with other Cas12 proteins and engineered Cas9 variants [41–45].

Several groups have investigated cleavage activity of the CasX family of enzymes [36,38,46] since the initial description by Burstein et al. [35]. Liu et al. [38] tested cleavage with CasX1 (DpbCas12e) and CasX2 (PlmCas12e) and determined that a gRNA with a 20 nt spacer cleaved 12–14 nt after the PAM on the NTS, and 22–25 nt after the PAM on the TS, generating a 5’ overhang from 7 to 14 nt lengths. They observed no single-stranded nuclease activity, unlike what is reported for Cas12a. Subsequently, Selkova et al. [46] tested the locations of the DpbCas12e staggered cleavage cuts using spacers of various lengths and employed high throughput sequencing to identify cleavage sites. Contrary to findings by Liu et al. [38], this group observed cleavage of the NTS at 17–19 nt from the PAM, and after the 22 nt on the TS, producing shorter 5’ overhangs of only 3–5 nt lengths. However, they also observed that shortening the spacer from 20 nt to 16 nt shifted the cleavage site on the NTS closer to the PAM, producing longer 5’ overhangs of 6–8 nts in length. In a recent study by Tsuchida et al. [36], homologs of CasX and single guide RNAs (sgRNA) were found to impact cleavage efficiency of these editors. This group observed enhanced *in vitro* cleavage of a DNA target with DpbCas12e compared to PlmCas12e, but improved cleavage with PlmCas12e in cells suggesting cell-free *in vitro* cleavage activity is impacted by the purity of the recombinant enzyme. Further, they determined that three nucleotide-binding loops within CasX may enhance PAM-proximal region recognition, spacer interaction, and DNA substrate loading, all of which may contribute to the different cleavage efficiencies among homologs of Cas12e [36].

To further understand factors influencing CasX2 function, we performed a systematic analysis of *CCR5* gene cleavage by multiple gRNAs of varying spacer lengths in a cell-free *in vitro* model. We also analysed the impact of changes in the fourth nucleotide of the PAM, (the ‘N’ in TTCN), to determine the extent to which CasX2 exhibits any base preference at this site. Our findings showed that cleavage efficiencies varied with both the DNA target site, the length of the spacer, and the nucleotide in the fourth position of the PAM. Although these studies were carried out in a cell-free *in vitro* assay, we expect that they will have relevance for CRISPR/Cas gene editing approaches designed for cell model systems or for patient therapeutics. These findings underscore the need for a more systematic analysis of CasX2 target selection and gRNA design, with the goal to improve therapeutic implementation of these new CRISPR enzymes.

## Methods

### Recombinant CasX2 protein expression and purification

PlmCas12e (CasX2) expression plasmids were generated by ligation independent cloning. The CasX2 open reading frame (ORF) was amplified from the plasmid pBLO 62.5 (pBLO 62.5 was a gift from Jennifer Doudna and Benjamin Oakes, Addgene plasmid #123124; http://n2t.net/addgene:123124; RRID:Addgene 123,124) [38]. The amplicons containing CasX2 were subcloned into the pET His10 MBP Asn10 TEV LIC cloning vector (pET His10 MBP Asn10 TEV LIC cloning vector (2CT–10) was a gift from Scott Gradia, Addgene plasmid #55209; http://n2t.net/addgene:55209; RRID: Addgene_55209), and verified through Sanger sequencing. The final plasmid expressed CasX2 with a tobacco etch virus (TEV)-cleavable N-terminal His10-MBP fusion tag.

Histidine (His)10-maltose binding protein (MBP)-CasX2-2CT-10 was transformed and expressed in Rosetta™(DE3) Competent Cells (Novagen, St. Louis, MO). Cells were grown at 37°C in 1 litre of Luria-Bertani (LB) broth supplemented with ampicillin (100 µg/ml), chloramphenicol (34 µg/mL), and 0.1% glucose. After growing to an OD_600_ of 0.5, the temperature was decreased to 16°C, and CasX2 expression induced with the addition of 0.5 mM isopropyl β-D-1 thiogalactopyranoside (IPTG, EMD Millipore, Burlington, MA). After 16 hours at 16°C, bacterial cells were pelleted and resuspended in lysis buffer (50 mM HEPES, pH 7.5, 0.5 M NaCl, 0.5 mM tris-(2-carboxyethyl)-phosphine (TCEP), 20 mM imidazole, 10% (v/v) glycerol, 1 mM phenylmethyl-sulphonyl fluoride (PMSF), 1 tablet protease inhibitor cocktail (Sigma Aldrich, St. Louis, MO) per 50 mL of solution and 0.5 mg/mL lysozyme), at a volume of 5 mL lysis buffer per gram of pellet. The resuspended cells were disrupted by sonication, and cellular debris removed by ultra-centrifugation for 30 minutes at 35,000 × g. The supernatant was loaded onto a 5 mL HisTrap HP cartridge (#17524802, Cytiva Life Sciences, Marlborough, MA) using an AKTA Go Chromatography system (#29383015, Cytiva Life Sciences). Columns were washed with 50 mL nickel (Ni) wash buffer 1 (50 mM HEPES, pH 7.5, 0.5 M NaCl, 0.5 mM tris (2-carboxyethyl) phosphine (TCEP), 20 mM imidazole, 10% (v/v) glycerol) and 50 mL Ni wash buffer 2 (50 mM HEPES, pH 7.5, 0.6 M NaCl, 0.5 mM TCEP, 30 mM imidazole, 10% (v/v) glycerol), and eluted in 1.5 mL fractions with 15 mL Ni elution buffer (50 mM HEPES, pH 7.5, 0.5 M NaCl, 0.5 mM TCEP, 400 mM imidazole, 10% (v/v) glycerol). Fractions containing the peak elution products (as determined by spectrophotometry) were combined, and diluted in 2× MBP wash buffer (50 mM HEPES, pH 7.5, 0.5 M NaCl, 0.5 mM TCEP, 10% (v/v) glycerol). To further purify CasX2, the diluted Ni column elution was subjected to a gravity fed column packed with 4 mL amylose resin (# E8021, New England Biological, Ipswich, MA), washed with 50 mL of MBP wash buffer and eluted in 1 mL fractions with MBP elution buffer (50 mM HEPES, pH 7.5, 0.5 M NaCl, 0.5 mM TCEP, 10% (v/v) glycerol, and 20 mM maltose). The eluted samples were visualized via sodium dodecyl sulphate-polyacrylamide gel electrophoresis (SDS-PAGE), and fractions containing the target product were combined and dialysed overnight at 4°C against storage buffer (50 mM HEPES, pH 7.5, 0.5 M KCl, 0.5 mM TCEP, and 20% (v/v) glycerol). The protein product was recovered, concentrated using 50 kDa molecular weight cut-off Amicon Ultra Centrifugation Filter units (Millipore, Burlington, MA), and stored in aliquots at −80°C.

### Design of gRNAs

Human *CCR5* (NM_001394783.1), located on chromosome 3, was selected as the target sequence for CasX2 cleavage. Single guide RNAs (sgRNAs) were designed using Benchling (www.benchling.com) using a custom 5’ TTCN PAM sequence. Ten spacer sequences were selected with the highest possible specificity scores (all were >92.0), with five sites located upstream and five sites located downstream of the *CCR5-Δ32* region within exon 2 of CCR5 (Chr3:46372947–46373940). The specificity score (or ‘off target score’) is generally dependent on a positional weight matrix that takes into account tolerance for mismatches at specific positions and, for newer approaches, tolerance for specific mismatches. However, methods to calculate such variables do not exist for Cas12e. Thus, the specificity scores were determined through gRNA design and selection using the CRISPR Tools in Benchling. These tools were used because they allowed for the use of a custom PAM at the 5’ end and are, therefore, compatible with Cas12e. The approach is a modified version of the ‘MIT Score’ described in Hsu et al. [47]. In Benchling, the default settings with custom PAMs were to score gRNAs without using positional weighting [47]. The resulting score is therefore less an indication of the likelihood of on-target versus off-target activity, than a simple scoring based on the number of sites in the human genome with PAM and up to 4 mismatches.

### In vitro *T7 single guide (sg)RNA transcription*

Transcription templates for sgRNAs were constructed with a 5’ T7 promoter and the CasX2 scaffold sequence followed by the selected 17–23 nt spacer sequences complementary to the target DNA sequence. The template was generated through overlap extension polymerase chain reaction (PCR) using primers that were purchased from Integrated DNA Technologies (IDT, Coralville, IA). An initial annealing step was performed with a 110 nt forward (F) primer, X2_F_template (5’-GAATTAATACGACTCACTATAGTACTGGCGCTTTTATCTCATTACTTTGAGAGCCATCA

CCAGCGACTATGTCGTATGGGTAAAGCGCT-3’), containing the sequences for the T7 promoter and the tracr region, and a reverse (R) primer, X2_R_template (5’-(17–23 bp spacer) + CTTTGATGCTTCTTATTTATCGGATTTCTCTCCGATAAATA-3’) containing the various spacer sequences. This reaction combined 45 µM of the forward and reverse primers in 1× STE buffer (Qiagen, Germantown, MD) in a total volume of 10 µl, followed by annealing for 5 minutes at 95°C, and then slow cooled to room temperature before the addition of 90 µl of nuclease free-H_2_O. Post-annealing PCR was performed with the addition of a shorter forward primer (5’-GAAATTAATACGACTCACTATAGTACTGGCGCTTTTATCT-3’) and the X2_R_Template in a 10 µM final concentration with 5 µl polymerase master mix at the following cycling conditions: 98°C/30 seconds, 25 × (98°C/5 seconds, 68°C/10 seconds, 72°C/15 seconds), 72°C/2 minutes, followed by a hold at 4°C. The PCR product was then purified with the Qia-quick PCR clean up kit (Qiagen). The Hi-Scribe T7 High Yield RNA synthesis kit (NEB, Ipswich, MA) was used to generate RNA transcripts at 37°C for 16 hours, according to the manufacturer’s protocol. The final sgRNA sequence is: 5’-UACUGGCGCUUUUAUCUCAUUACUUUGAGAGCCAUCACCAGC

GACUAUGUCGUAUGGGUAAAGCGCUUAUUUAUCGGAGAGAAAUCCGAUAAAUAAGAAGAUCAAAG + (17–23 nt spacer)-3’. Sequences of the DNA oligonucleotides used for the *in vitro* transcription of the sgRNAs are shown in Supplemental Table 1. The RNA sequences of each of the spacers from 17 to 23 nts in length are shown in Supplemental Table S2.

**Table 1.** CCR5 sgRNA position on chromosome 3 and specificity score.

RNA Guide	Position	Strand	Specificity Score	PAM
1	46372957	(-)	93.552	TTCA
2	46373065	(-)	92.825	TTCA
3	46373164	(+)	93.068	TTCT
4	46373172	(-)	98.547	TTCT
5	46373178	(+)	98.657	TTCC
6	46373664	(+)	97.692	TTCT
7	46373680	(-)	95.179	TTCA
8	46373897	(+)	96.788	TTCT
9	46373904	(+)	98.825	TTCC
10	46373921	(-)	96.577	TTCC

### *DNA target generation for* in vitro *cleavage (IVC) reactions*

The 6,415 bp pcDNA3-CCR5 plasmid was a gift from Erik Procko (Addgene plasmid #98943; http://n2t/net/addgene:98943; RRID:Addgene_98943) [48]. Prior to use in IVC reactions, a smaller target fragment of 2,812 bp was generated by sequential digestion using the restriction enzymes SmaI and SpeI, and subsequent purification via Qia-Quick PCR clean up columns (Qiagen), according to the manufacturer’s protocol. The restriction digested DNA was purified and quantitated using a Qubit double-strand DNA assay.

### In vitro *cleavage reactions*

Cell-free IVC reactions were performed in a two-step process. First, 10 µl of a ribonucleoprotein complex (RNP) was formed by incubating each sgRNA and CasX2 at equimolar ratios (2 µM sgRNA +2 µM CasX2) in IVC reaction buffer (20 mM HEPES pH 7, 100 mM KCl, 5 mM MgCl_2_, 0.1 mM EDTA, 1% glycerol) at 37°C for 10 minutes. RNP:DNA target ratios from 10:1 to 80:1 were initially tested in IVC assays using gRNAs with a 20 nt spacer length and the native PAM. IVC reactions were incubated at 37°C for 60 minutes. Because we observed slight variations in cleavage activity when comparing one RNP to another in IVC reactions, the RNP to DNA target input was adjusted to maintain the cleavage reaction in a linear range, so that relative semi-quantitative densitometry imaging could be achieved for each gRNA. IVC reactions assessing the impact of spacer length and the type of nucleotide in the fourth position of the PAM were performed using a final concentration of target DNA between 5 and 6 nM in 20 µL reactions, and the appropriate volume of 2 µM RNP, to yield the desired RNP:target DNA ratios between 11.6:1 and 35:1. For sgRNAs 1, 3, 4, 7, 8 and 10, the molar ratio of RNP to target DNA was 19:1. For sgRNA 5 it was 11.6:1, for sgRNA 9 it was 16.5:1, for sgRNA 6 it was 21.5:1, and for sgRNA 2 it was 35:1. Gel electrophoresis using a 1% agarose gel was performed to visualize IVC reaction products. Cleavage products on the gels were visualized using a Bio-Rad ChemiDoc MP imaging system and the percent cleavage of the target sequence was determined via densitometric analysis using Bio-Rad Imaging Lab software (V6.1.0 Build7–2020).

### *Design of modified* CCR5 *target sequences with altered PAM sequences*

Four separate gene fragments (gene blocks, gBlocks) corresponding to a 1,114 bp region of the human *CCR5* gene sequence were synthesized by IDT. For each of these four gBlocks, the fourth nucleotide in the PAM for nine of the ten gRNA target regions was changed to either an adenine (A), cytosine (C), guanine (G) or thymidine (T), resulting in four distinct target sequences that differed only in the fourth nucleotide of the PAM. Each of the four gBlocks were cloned separately into the plasmid pcDNA3. Briefly, the gBlocks and the plasmid DNA were digested with the NheI and XhoI restriction enzymes, and ligated with T4 DNA ligase in a 3:1 molar ratio before transforming into NEB® 5-alpha competent *E. coli*. Selected colonies were screened and verified by Sanger Sequencing.

## Results

### *Cleavage activity of gRNAs targeting* CCR5

**Figure 1.**
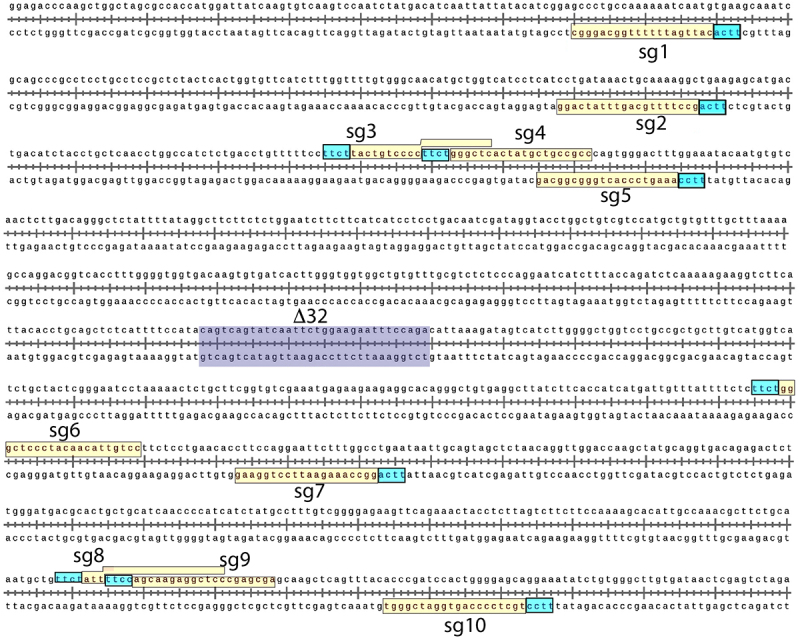
Target sequences for gRNAs within the human CCR5 gene. Schematic of the locations of the target sequences for gRNAs (20 base protospacer sequence; yellow highlighting) and PAM sequences (light blue highlighting) relative to the location of the region that would be deleted in the Δ32 mutation (purple highlighting), within a 1,000 base pair segment of exon 2 of the human CCR5 gene located on chromosome 3.

Ten different gRNAs with specificity to exon 2 of *CCR5* (Chr3:46372947–46373940) were selected to assess cleavage activity of PlmCas12e using a cell free *in vitro* cleavage reaction assay (Table 1). The gRNAs, referred to as single guide (sg) RNA 1, 2, 4, 7 and 10, bound to the (-) target DNA strand, while sgRNAs 3, 5, 6, 8 and 9 bound to the (+) target DNA strand. Specificity scores, as determined in Benchling.com and based on Hsu et al. [47], ranged from 92.825 (sgRNA 2) to 98.825 (sgRNA 9). Three gRNAs used a PAM sequence of TTCA, three used a PAM sequence of TTCC, and four used a PAM sequence of TTCT. The gRNAs were selected based on their location relative to the *CCR5-Δ32* site, with five gRNAs upstream of the *CCR5-Δ32* site, and five gRNAs downstream of the *CCR5-Δ32* site. Complete sequence information for the *CCR5* region of interest, gRNA binding sites, protospacer, and PAM locations are shown in Figure 1.

**Figure 2.**
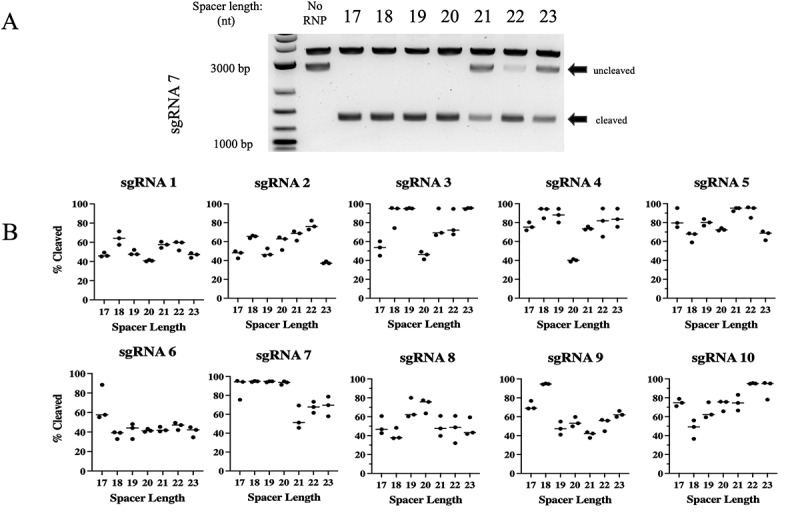
Cleavage activity of PlmCas12e is dependent on both gRNA length and target location.

**Note: (A)** Agarose gel separation was used to visualize PlmCas12e cleavage products after cell free *in vitro* cleavage. A *CCR5* target sequence of 2,812 nt was used to examine the effect of spacer length on DNA cleavage. A representative gel for sgRNA 7 is depicted as an example from which densitometry measurements were derived. The sgRNA 7 cleavage generated products of 1,368 nt and 1,444 nt in length.

A previous report by Selkova et al. [46] investigated the correlation between spacer length and location of the cleavage cuts on DNA targets. In their studies, they noticed slight differences in the efficiency of cleavage of one target as the lengths of the spacers were shortened. Based on their observation, we sought to carry out a systematic evaluation of spacer length on cleavage efficiency of ten different target sites to determine if a correlation existed between these variables. We used a 2,812 bp (Sma I/Spe I) fragment of *CCR5* derived from the plasmid pcDNA3-CCR5 that included all ten target sites. *In vitro* cleavage (IVC) reactions of the *CCR5* target were performed for all gRNAs with various spacer lengths from 17 to 23 nts, and each experiment was repeated for a total of three times with consistent findings. Molar ratios (MR) of RNP:target DNA were varied across gRNAs, in order to be able to assess the relative effects of changing the spacer length for each gRNA. As a result, cleavage activity can only be compared across spacer lengths within a target set and not across different targets. IVC reactions were then subjected to agarose gel electrophoresis, where cleavage of the target could be quantified. Figure 2a shows a representative experiment for sgRNA 7, and the results for the other nine gRNAs are shown in Supplemental Figure 1. We found that cleavage efficiencies varied with both the gRNA target site as well as spacer length. Although spacer length had an impact on the efficiency of cleavage for some gRNAs, we were unable to identify a consistent correlation between spacer length and cleavage activity across the 10 gRNAs we tested. For example, sgRNA 3 cut most efficiently at lengths of 18, 19 and 23 nts, sgRNA 7 cut most efficiently at lengths from 17 to 20 nt, and sgRNA 9 cut most efficiently at a length of 18 nt.

**(B)** Seven different spacer lengths from 17 nt to 23 nt for each of ten different gRNAs were analysed for *in* vitro cleavage activity of the 2,812 nt *CCR5* DNA target by agarose gel electrophoresis. Molar ratios (MR) of RNP:target of 11.6:1 were used for sg5, MR of 16.5: for sg9, MR of 19:1 for sg1,3,4,7,8 and 10, MR of 21.5:1: for sg6, and MR of 35:1 for sg2. The densitometry of the cleavage products was analysed for each gRNA at spacer lengths ranging from 17 to 23 nts, and the median percent cleavage of the target plotted in a dot blot using *Prism Soft Inc*. All experiments were run in triplicate.

Densitometry analysis was performed to determine the cleavage activity of each gRNA at all lengths examined. The median percentage of the target sequence cleaved was plotted against spacer length from all gRNAs and shown in Figure 2B. Characteristics of each gRNA were noted, including spacer length, GC content, PAM sequence, cutting efficiency and Gibb’s free energy (δG) (Supplemental Table 2). We assessed cleavage activity as a function of spacer length to determine whether some lengths were significantly more efficient than others for all target sites. A two-way ANOVA of cleavage activity as a function of gRNA length did not reveal significant differences in cleavage efficacy when *p* values were adjusted for multiple comparisons using Tukey’s honest significant differences; all *p* values exceeded 0.05.

### Impact of PAM sequence on gRNA cleavage activity

**Figure 3.**
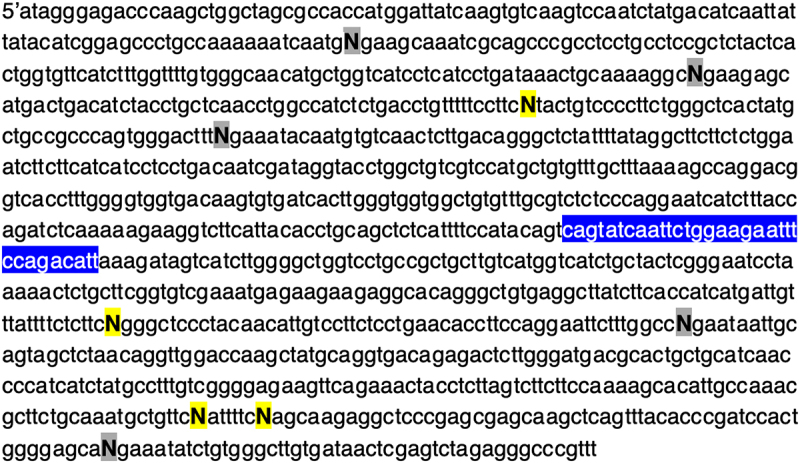
Location of nine terminal PAM bases that were changed to assess CasX2 PAM preference. Four different CCR5 gene fragments of 1,114 bp (gBlocks) were synthesized to include nine of the ten gRNA target regions. The terminal PAM base for each gRNA was changed to either an A, C, G or T in each gBlock. Each of the four gene fragments were separately cloned into the pcDNA3.1 vector and then restriction enzyme digested with NheI and XhoI to yield a 1,074 bp target. In the CCR5 gene region of interest, four PAM regions were located on the (+) strand and five PAM regions were located on the (-) strand. For clarity, only the (+) strand (5’→ 3”) of the CCR5 sequence is illustrated here, where a yellow highlighted N indicates a PAM terminal base on the (+) strand, and a grey highlighted N indicates a PAM terminal base on the (-) strand. The blue highlighted region is the wild-type sequence of CCR5 that would be deleted in the CCR5-Δ32 mutation. The sgRNAs 1, 2, 3 and 5 bind upstream of the CCR5-Δ32 region and sgRNAs 6, 7, 8, 9 and 10 bind downstream.

**Figure 4.**
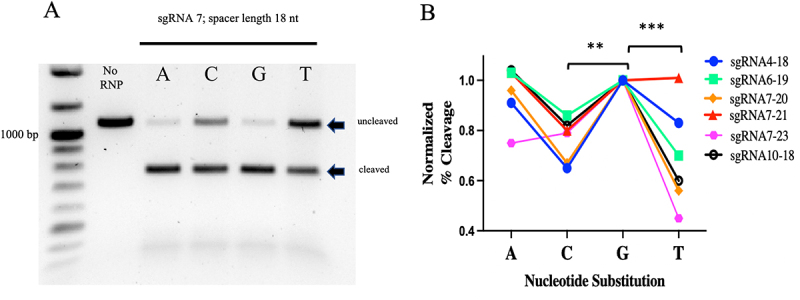
The terminal PAM base influences PlmCas12e cleavage activity.

Given that for individual gRNAs we observed differences in cleavage activity *in vitro* when the spacer lengths were varied, we next sought to determine whether the PAM sequence could also influence the performance of the sgRNA. Considering that the PAM for CasX2 is TTCN, the fourth nucleotide of the PAM can be any purine or pyrimidine. For these experiments, we synthesized four different target DNA sequences in which the fourth nucleotide of the PAM for each gRNA target was either an A, G, C or T nucleotide. Figure 3 depicts the location of each of the modified bases in the target, where ‘N’ represents the fourth nucleotide in the PAM for each of the gRNAs. Four substitutions were located upstream of the *CCR5-Δ32* location, and five substitutions were located downstream. One gRNA was not evaluated (sgRNA 3) because the PAM for sgRNA 4 was located within the sgRNA 3 protospacer and would have created a mismatch between sgRNA 3 and its target. We chose several sgRNAs at varying spacer lengths to assess cleavage of each of the four DNA targets. We observed that for each gRNA, cleavage activity varied dramatically according to the terminal PAM base in the target DNA. For example, sgRNA 7 at a length of 18 nt cleaved target DNA more efficiency when the PAM sequence ended with either an A or G, compared to the same sgRNA when the target had a terminal PAM base of C or T (Figure 4A). This finding was consistent for many of the gRNAs tested regardless of spacer lengths and indicated that a purine in the terminal position of the PAM was generally preferred for target cleavage. Normalized percent cleavage from six different biological replicates is shown in Figure 4B. Normalization was performed due to the variation in cleavage activity among the four targets, and given that our primary goal was only to assess the impact of changing the fourth nucleotide in the PAM (TTC***N***). As we observed the highest cleavage efficiencies with most gRNAs when the PAM was TTCG, we set the percentage cleavage efficiency with each RNP using a target with the PAM of TTCG to a value of 1.0, and divided cleavage percentages for the other PAM targets by the percentage observed with TTCG. Additional replicates with other gRNAs and spacer lengths are shown in Supplemental Figure 2. When we examined the differences in PlmCas12e cleavage comparing the type of fourth nucleotide in the PAM via linear modelling, the analysis revealed that for percent cleavage as a function of gRNA binding location, gRNA length, and fourth PAM nucleotide base, both C and T are significantly less effective than G (e.g. C (*p* < 0.01), and T (*p* < 0.001)).

**A)**. *CCR5* gene fragments (1,074 nt) were generated with different terminal PAM bases of either A, C, G or T. These four DNA targets were assessed for cleavage activity by sgRNA 7 at a length of 18 nt. Cleavage products were then run on a 1% agarose gel so that differences in the cleavage activity for each target could be quantified. A representative experiment is shown in panel A. In triplicate experiments, cleavage activities for sgRNA 7 at length of 18 nt, were highest for targets with a terminal ‘G’ (median cleavage 88.5%), followed by those with a terminal ‘A’ (66.3%), a terminal ‘C’ (59.4%), and a terminal ‘T’ (43.6%).

**B)**. Cleavage activity for each terminal PAM base substitution was determined by gel densitometry and normalized to the ‘G’ target. Normalization to the TTCG target was performed to allow comparisons for each sgRNA for all targets. Results from four different sgRNAs across multiple spacer lengths show that purines A and G as the terminal PAM base demonstrated greater cleavage activity compared to pyrimidines C and T. Linear modelling reveals that for percent cleavage as a function of gRNA, spacer length and terminal PAM base, both C and T are significantly less than G (**p < 0.01, ***p < 0.001).

## Discussion

We sought to identify parameters associated with increased efficiency of PlmCas12e cleavage of DNA targets that would aid in the design and development of future gRNAs for Cas12e applications. The parameters we tested using a cell-free *in vitro* cleavage assay included target location, spacer length, and varying the fourth nucleotide in the PAM. Our results demonstrated that both spacer length and the fourth base in the PAM are important factors to consider when designing PlmCas12e gRNAs for gene editing.

The CRISPR/CasX2 (PlmCas12e) system, first identified from metagenomic sequencing of DNA extracted directly from natural microbial communities [35], uses a gRNA and tracrRNA complex to bind to the Cas enzyme. This ribonucleoprotein complex then cleaves a DNA target dictated both by the complementarity of the spacer to the target and recognition by the Cas enzyme of the PAM sequence. The Cas12e PAM, TTCN, was initially characterized using a gRNA with a 20 nt spacer length and did not resolve a strong preference at the fourth position [35]. However, a careful evaluation of the data obtained by Burstein et al., in their *E. coli* PAM depletion assay using the highest depletion value threshold reported, does indicate a slight preference for a purine at the fourth position for Cas12e [35]. Our results suggest that further consideration should be given to the PAM sequences of Cas12e, and that PAM preferences should be assessed using multiple gRNA/target DNA pairs in order to fully understand parameters influencing cleavage efficiency.

In addition to the PAM sequence, it was clear that other factors are likely to influence cleavage efficiency. Unlike the well-studied Cas9 systems, Cas12e editors deliver a cleavage cut on the target DNA that is asymmetrical. The precise location of the cleavage cuts on the TS and NTS, and the length of the 5’ overhang, appear to vary depending on the specific gRNAs and target DNA as reported by Liu et al., and Selkova et al. [38,46]. Although their investigations focused on defining changes in the location of the cleavage events with variation in the length of the spacer, some effect on cleavage activity for DpbCas12e was also noted. Our studies, in which we undertook a comprehensive analysis of cleavage activity by varying the spacer length across multiple target sites, clearly show that cleavage activity is dependent on multiple factors. Future studies should directly compare our findings using PlmCas12e with the DpbCas12e studies of Selkova et al. [46], to determine whether the impact of spacer length on activity, and location of the TS and NTS cleavage sites, are conserved between these two CasX proteins. Regardless, it is clear that spacer length is an important variable to consider when testing Cas12e-based approaches. Optimization of spacer length may impact activity, specificity, and the location of the cut sites; information that would be important to consider when the goal is not only to remove a particular target region but also to promote cellular repair mechanisms such as homology directed repair. Thus, to create the *CCR5-Δ32* mutation in *CCR5* in haematopoietic stem cells, testing of different gRNA pairs at varying spacer lengths and target sites are likely to be critical factors to be optimized.

We undertook a systematic analysis of the impact of guide RNA length on cleavage activity, using a portion of the *CCR5* gene that included the area deleted in the *CCR5-Δ32* mutation as a target region. Ten different gRNAs were designed, and each gRNA was transcribed and tested at varying lengths from 17 to 23 nts, and used in cell free *in vitro* reactions where cleavage activity could be experimentally measured. Our findings demonstrated that cleavage activity varied depending on spacer lengths for each particular gRNA, but there was no consistent pattern among the different gRNAs. Some of the gRNAs, including sgRNA 1, sgRNA 5 and sgRNA 6, expressed consistent levels of cleavage regardless of spacer length. Others, including sgRNA 2 and sgRNA 10, showed increased cleavage efficiency as spacer length increased, whereas sgRNA 7 and sgRNA 8 showed declining cleavage efficiencies as spacer length increased. We observed remarkable consistency with repeated biological replicates, suggesting that these findings are particular for the gRNAs we tested. The wide range of cleavage activity for particular guide RNAs observed as the spacer lengths changed also indicated that factors other than gRNA binding location, or DNA strand location, were impacting Cas12e cleavage events. Our findings clearly indicate that careful gRNA design and testing of a wide range of spacer lengths is important for selecting optimal gRNAs for DNA cleavage and are likely to be important for testing gRNAs in a cell model system.

We next assessed whether the fourth nucleotide in the PAM could impact cleavage activity of the gRNAs. As CasX recognizes the sequence TTCN, it should cleave DNA targets adjacent to a PAM of TTCA, TTCG, TTCT and TTCC. To determine the contribution of the PAM sequence, we designed four different gBlock targets, substituting the fourth nucleotide in each PAM region with either an A, G, T or C. For these experiments, we chose several of the gRNAs at varying lengths. Our results showed that, in general, PAM sequences that ended in a purine (TTCA and TTCG) demonstrated enhanced cleavage over PAM sequences ending in a pyrimidine (TTCT and TTCC). This finding suggests that in addition to the gRNA sequence and the gRNA length, the sequence of bases that comprise the PAM also factor into cleavage efficiency. Because cleavage mediated by PlmCas12e is distal to the PAM, it is likely that the purine–purine bond adjacent to the gRNA binding site is not directly implicated in cleavage by the RuvC domain of CasX2, but to other, as of yet, undefined factors. Thus, our findings indicate that for PlmCas12e, considerations of PAM sequence at presumptive target sites as well as testing various spacer lengths should be considered when designing gRNAs for specific gene cleavage. For gRNA pair selections, the complementarity of the 5’ overhangs can also be considered to promote homology-directed repair.

The studies reported in this manuscript addressed the optimal cell free *in vitro* cleavage conditions for the novel PlmCas12e editor, CasX2. We chose to use *CCR5* as a target because of the importance of this receptor in HIV-1 infection. Our goal is to design guide pairs that flank the area of *CCR5* gene that includes the *CCR5-Δ32* mutation in such a way that we can insert a donor fragment of DNA expressing the *CCR5-Δ32* mutation to replace the wild-type sequence in haematopoietic stem cells. Future studies should focus on understanding the applicability of these findings to CasX2 cleavage activity and specificity in cells, where cellular repair mechanisms as well as other factors may impact cleavage activity. Understanding PAM preferences and optimal gRNA lengths will assist us in designing a therapeutic with a higher probability of success.

Based on our findings and those of others, we believe that this type of therapeutic approach can be best accomplished using novel Cas12e CRISPR systems. Advantages to using Cas12e editors include lack of pre-existing immunity to Planctomycetes, the bacterial species from which PlmCas12e derives, the smaller size of Cas12e editors compared to *Sp*Cas9, and the staggered cleavage cuts that will allow for insertion of donor DNA sequences with the use of gRNA pairs. Treatment of bone marrow-derived haematopoietic stem cells (HSCs) *ex vivo* would have several advantages over *in vivo* delivery of Cas enzyme and gRNA. An *ex vivo* approach would increase the editing frequency of HSC because the calibration of CRISPR/Cas to target cells can be achieved. Further, *ex vivo* treatment would allow for the selection of gene-edited cells prior to infusion. We have developed a mathematical model to mimic the therapeutic potential of CRISPR/Cas gene editing of HSCs to produce a CCR5^null^ immune system, including in the presence of replicating HIV-1 [49], and have demonstrated the potential of a CCR5^null^ immune system to be resistant to HIV-1 infection. The *in vivo* delivery, either via ribonucleoproteins (RNPs) or by viral vector delivery, is another potential therapeutic approach that could be developed for delivery of Cas12e and gRNA pairs for *CCR5* editing. *In vivo* delivery of adeno-associated viral (AAV) vectors carrying the *Sa*Cas9 and gRNA pairs to excise the integrated HIV genome is currently undergoing a Phase 1/2 clinical trial (NCT05144386). Although a thorough analysis of repeated dosing with Cas enzyme *in vivo* has not been performed to date, it is likely that pre-exisisting immunity and recall immune reactions to these bacterial-derived proteins would be less likely from a non-pathogenic bacterium compared to common pathogens such as *Staphylococcus* or *Streptococcus*.

Our findings with respect to PlmCas12e spacer length and PAM sequence will contribute to the continued development of CasX-based CRISPR systems as a novel platform for genome editing for a variety of viral infections and genetic mutations causing human disease. The small size of PlmCas12e, dissimilar PAM, immunological novelty, and unique cleavage pattern differentiate this enzyme from Cas9, increasing therapeutic opportunities that are likely to offer important advantages compared to existing genome editing approaches.

## Supplementary Material

Supplemental Material

## Data Availability

The data that support the findings of this study are included in the manuscript.
