# Supplementary figures and images for: PlmCas12e (CasX2) cleavage of CCR5: impact of guide RNA spacer length and PAM sequence on cleavage activity
Source: RNA Biol. 2023 Jun 7;20(1):296–305. doi: 10.1080/15476286.2023.2221510 (PMC10251783; doi:10.1080/15476286.2023.2221510)

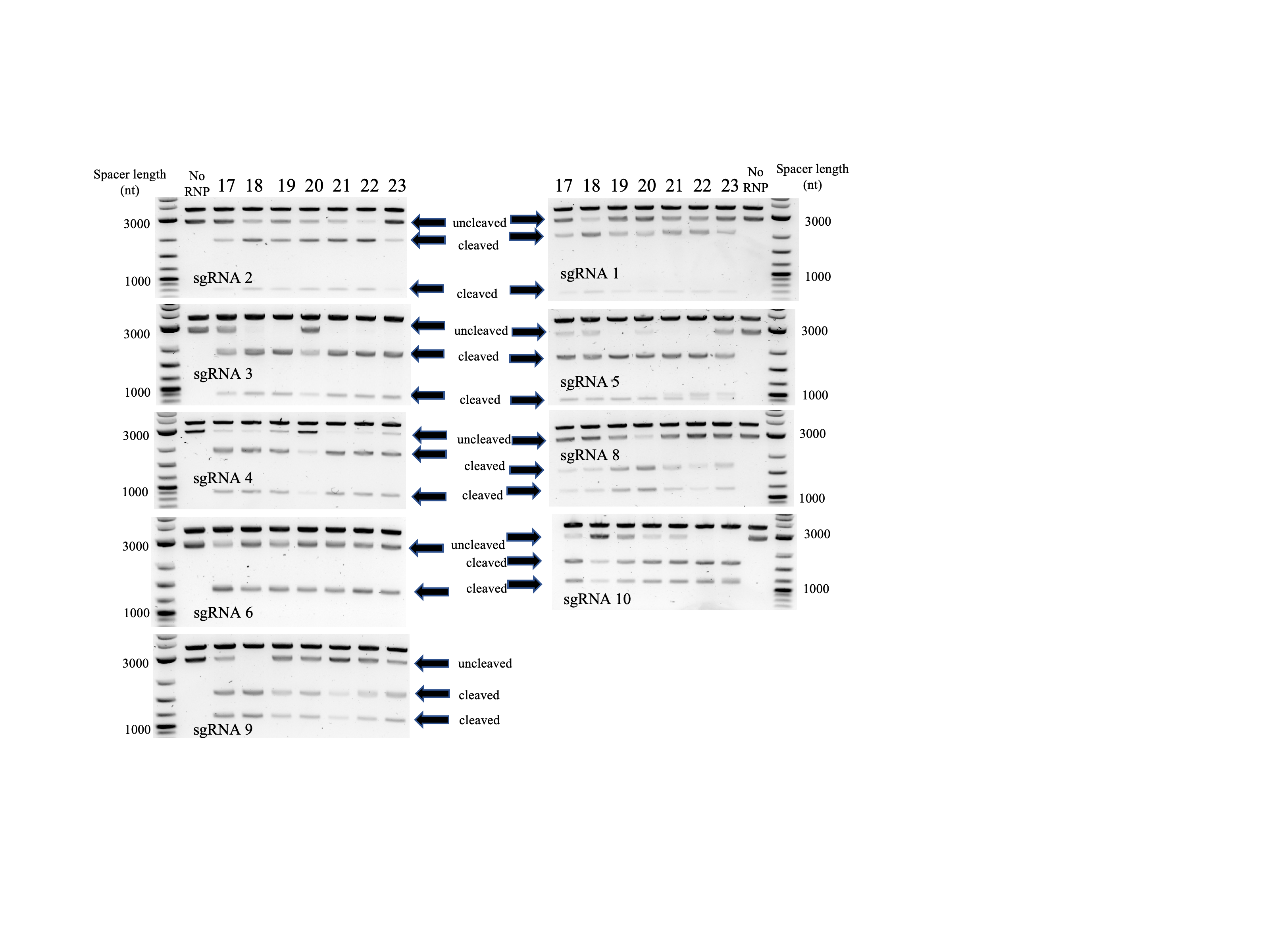

Supplement: Supplemental Material [file KRNB_A_2221510_SM2253.zip › Supplemental_Figure_1.tiff]

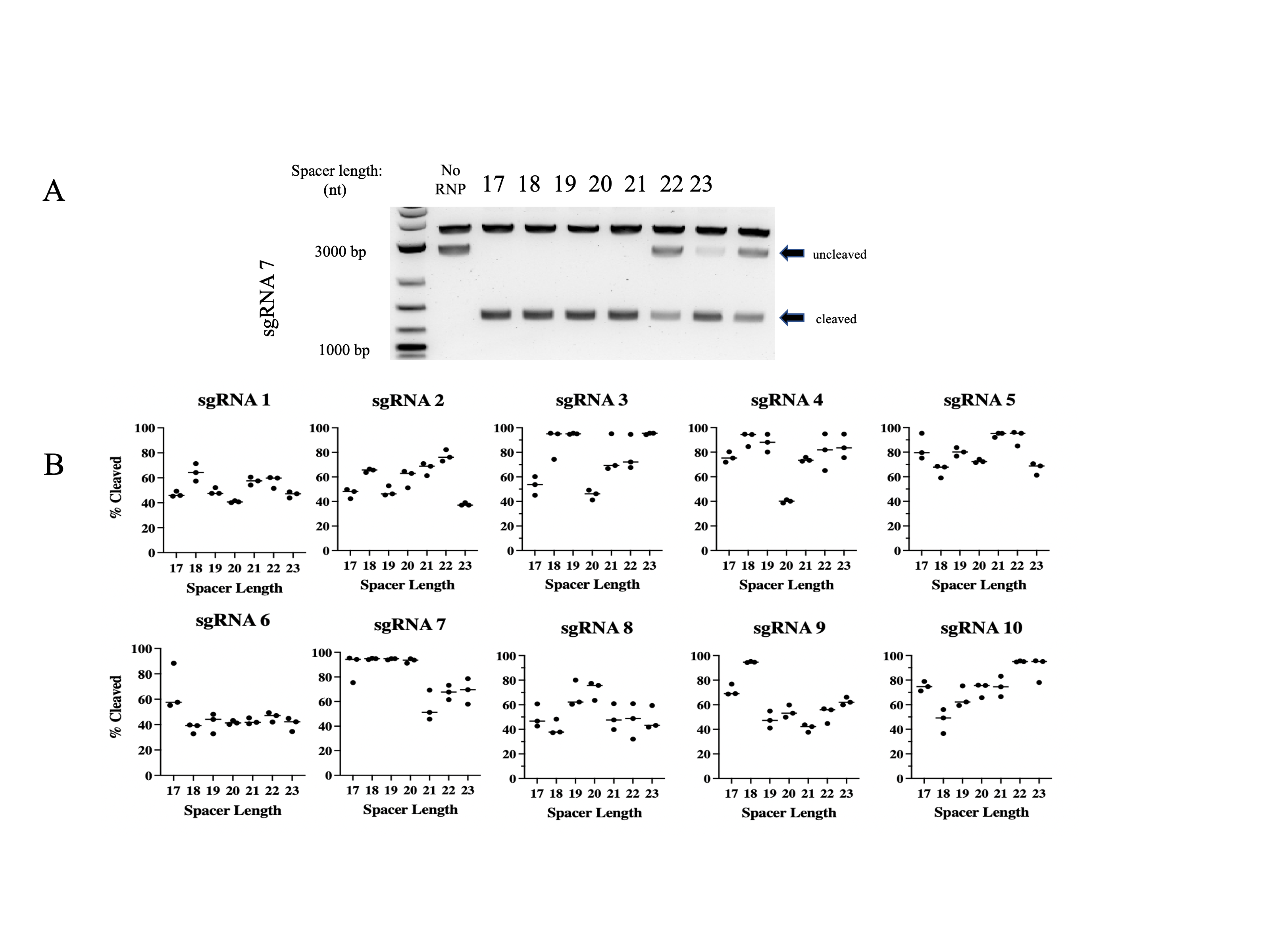

Supplement: Supplemental Material [file KRNB_A_2221510_SM2253.zip › Supplemental_Figure_2.tiff]
